# Supplementary material for: Prognostic Impacts of D816V KIT Mutation and Peri-Transplant RUNX1–RUNX1T1 MRD Monitoring on Acute Myeloid Leukemia with RUNX1–RUNX1T1
Source: Cancers (Basel). 2021 Jan 18;13(2):336. doi: 10.3390/cancers13020336 (PMC7831332; doi:10.3390/cancers13020336)

Figure S1. Trial profile.

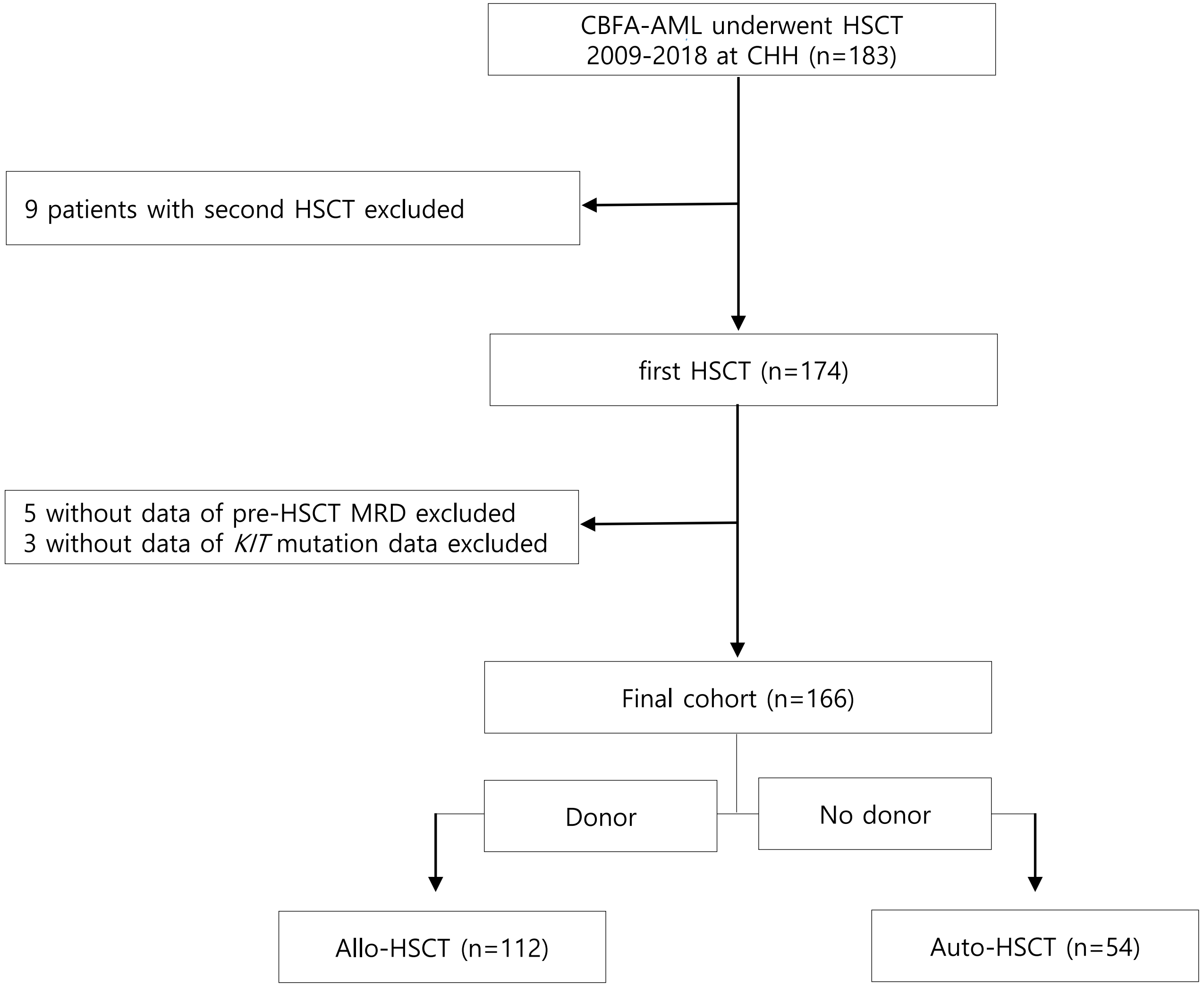

Figure S2. Cumulative incidence of relapse (CIR) according to types of *KIT* mutations. (A) CIR according to D816V *KIT* mutation, (B) D816Y *KIT* mutation, (C) D816H *KIT* mutation, (D) N822K *KIT* mutation, and (E) exon 8 *KIT* mutations.

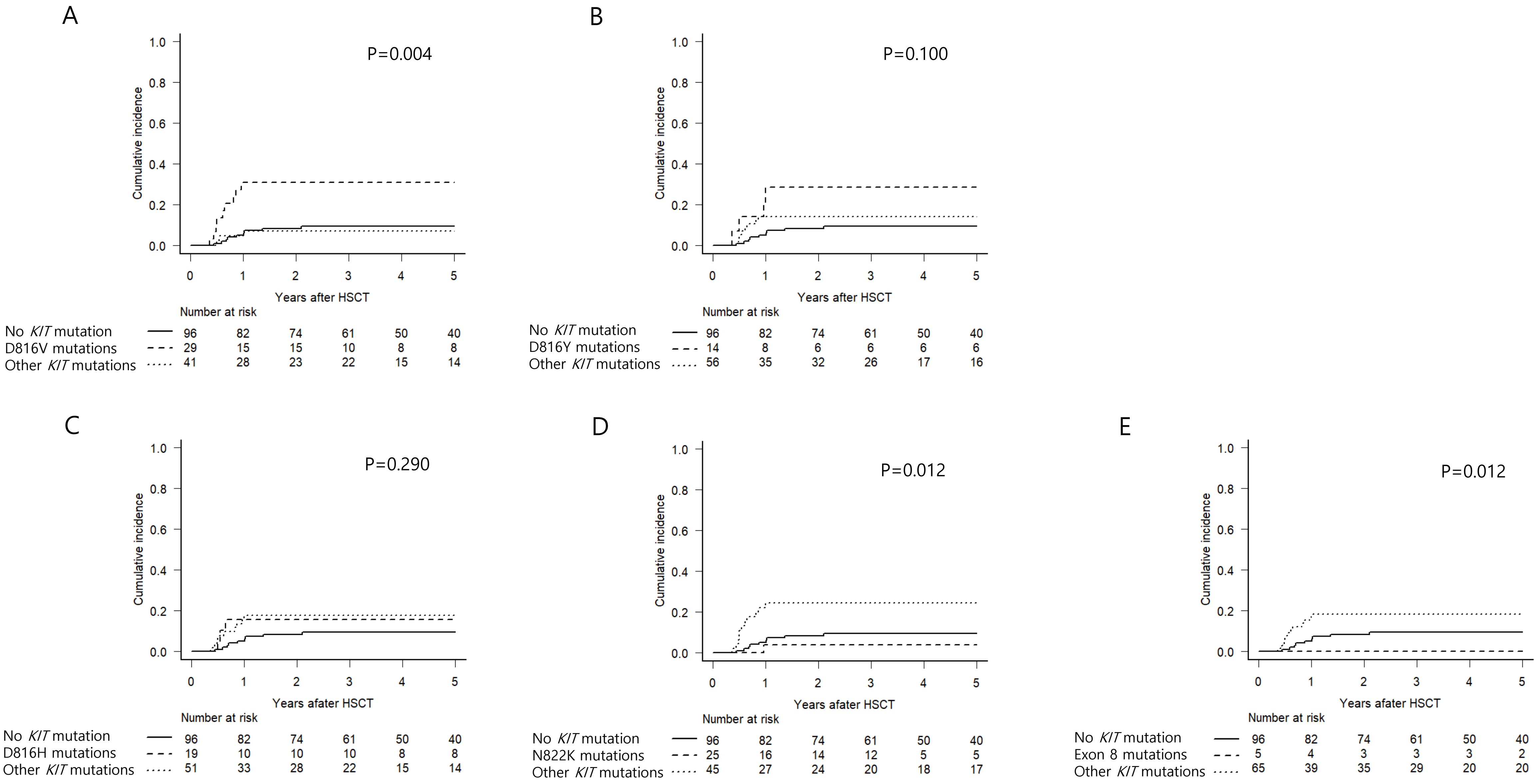

Figure S3. Survival outcomes according to D816V *KIT* mutation. (A) Non-relapse mortality, (B) disease-free survival, and (C) overall survival.

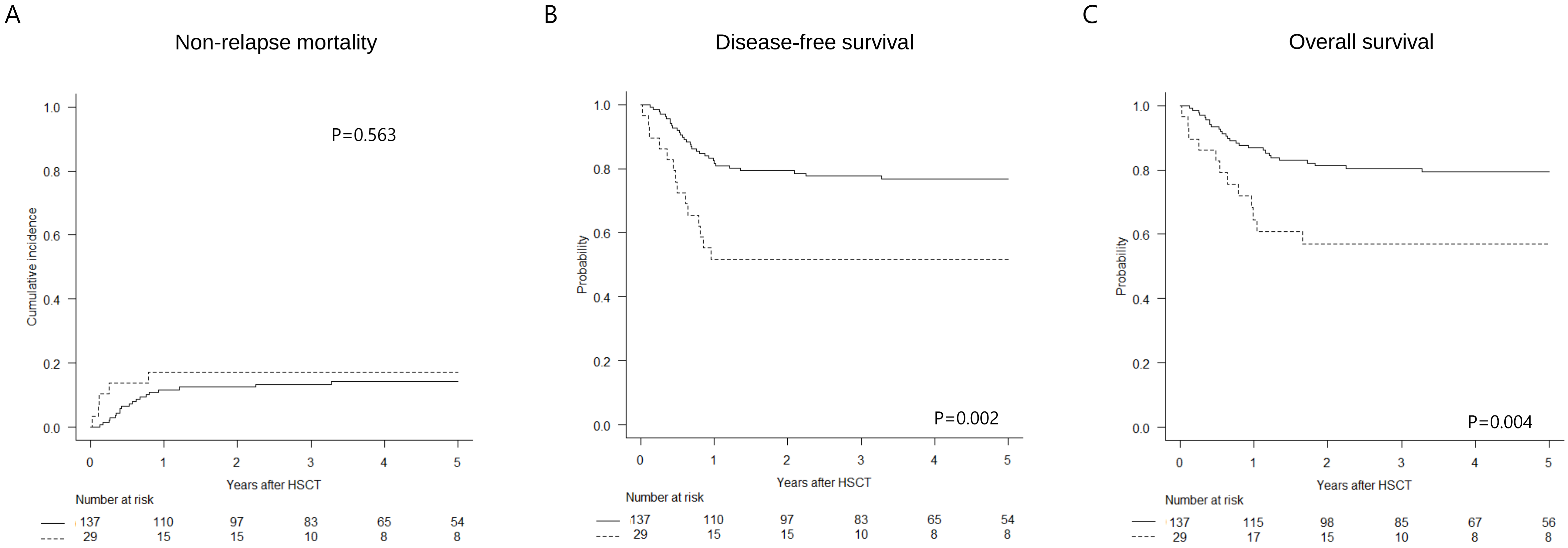

D816V *KIT* unmutated ——— D816V *KIT* mutated - - - -

Figure S4. Cumulative incidence of relapse according to various cutoffs of *RUNX1-RUNX1T1* transcript levels at pre-HSCT (A) and at 1 month (B) or 3 months (C) after HSCT.

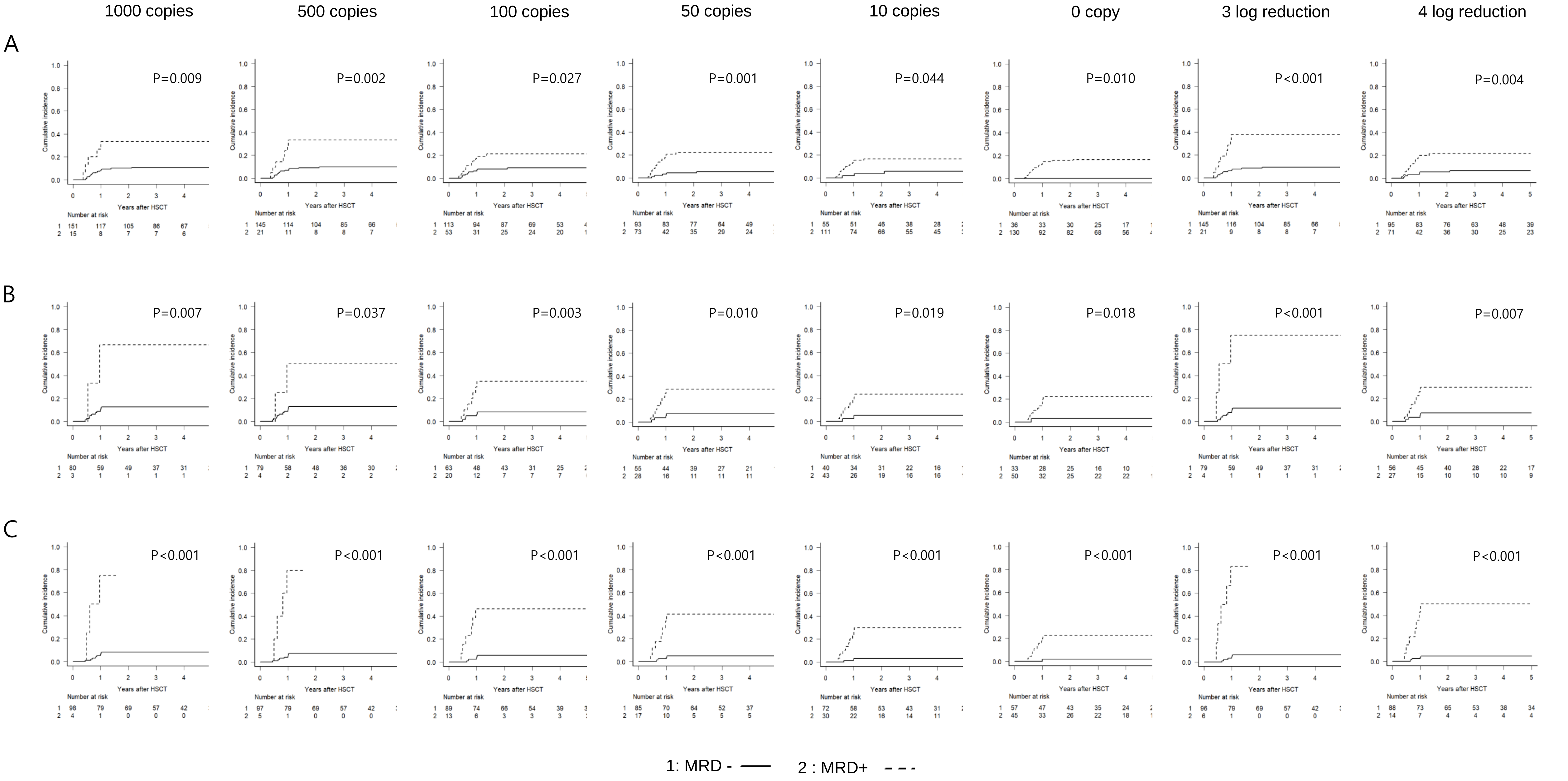

Figure S5. Survival outcomes according to MRD positivity defined by 3 log reduction of *RUNX1-RUNX1T1* levels at each time point. (A) Pre-HSCT and (B) at 1 month or (C) 3 months after HSCT.

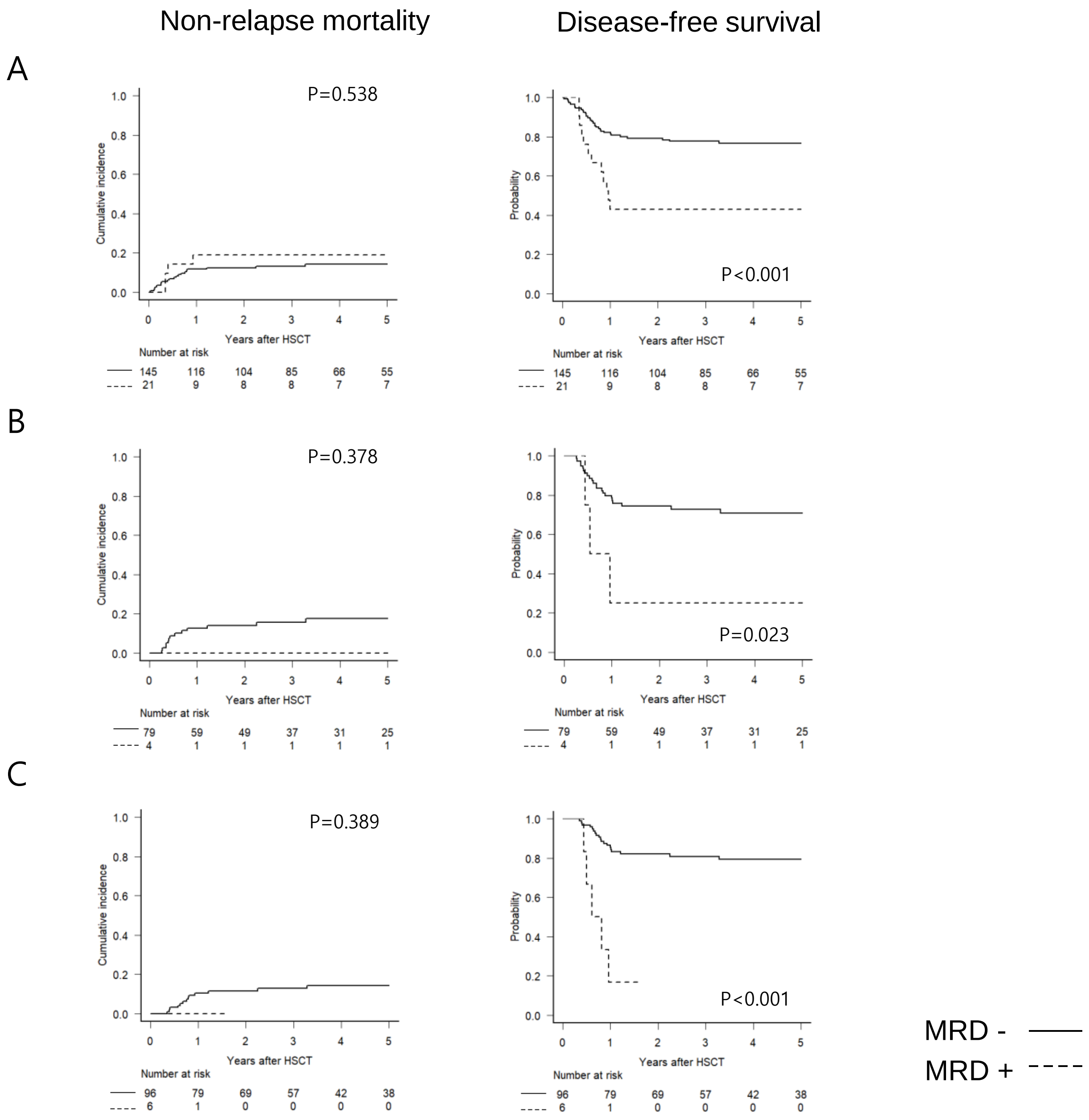

Figure S6. Survival outcomes according to transplant type. (A) Relapse, (B) non-relapse mortality, (C) disease-free survival and (D) overall survival.

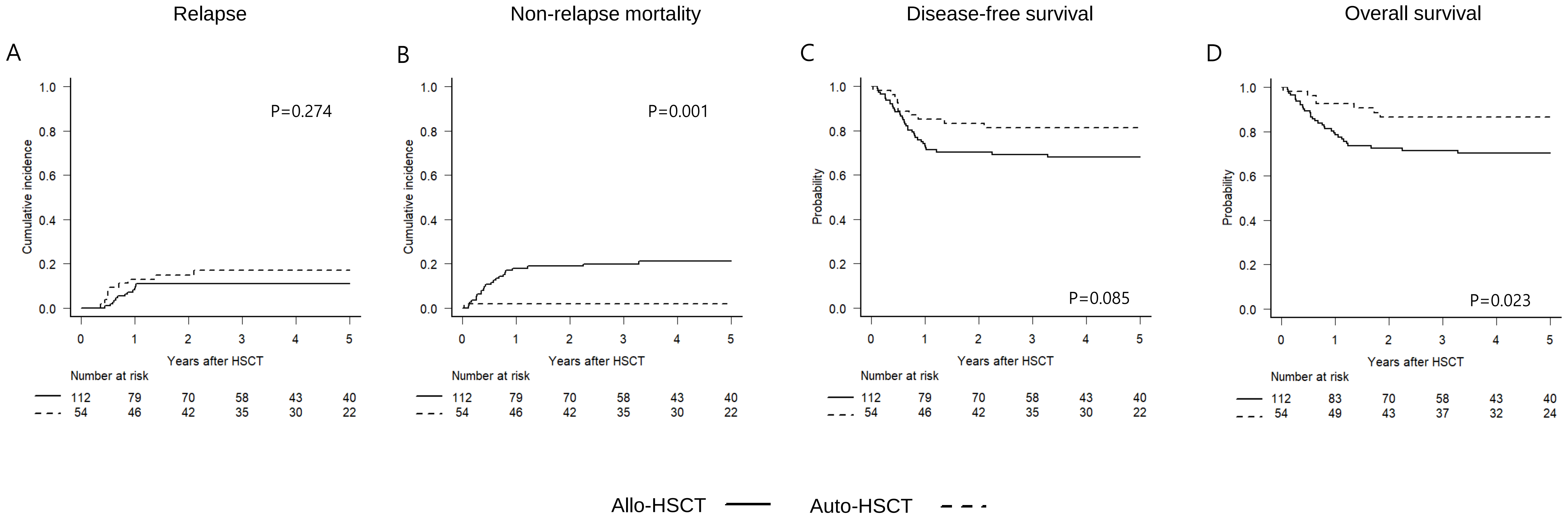

Figure S7. Survival outcomes in each group stratified by both D816V *KIT* mutations and the MRD positivity defined by 3 log reduction of *RUNX1-RUNX1T1* levels at pre-HSCT. (A) Non-relapse mortality, (B) disease-free survival, and (C) overall survival.

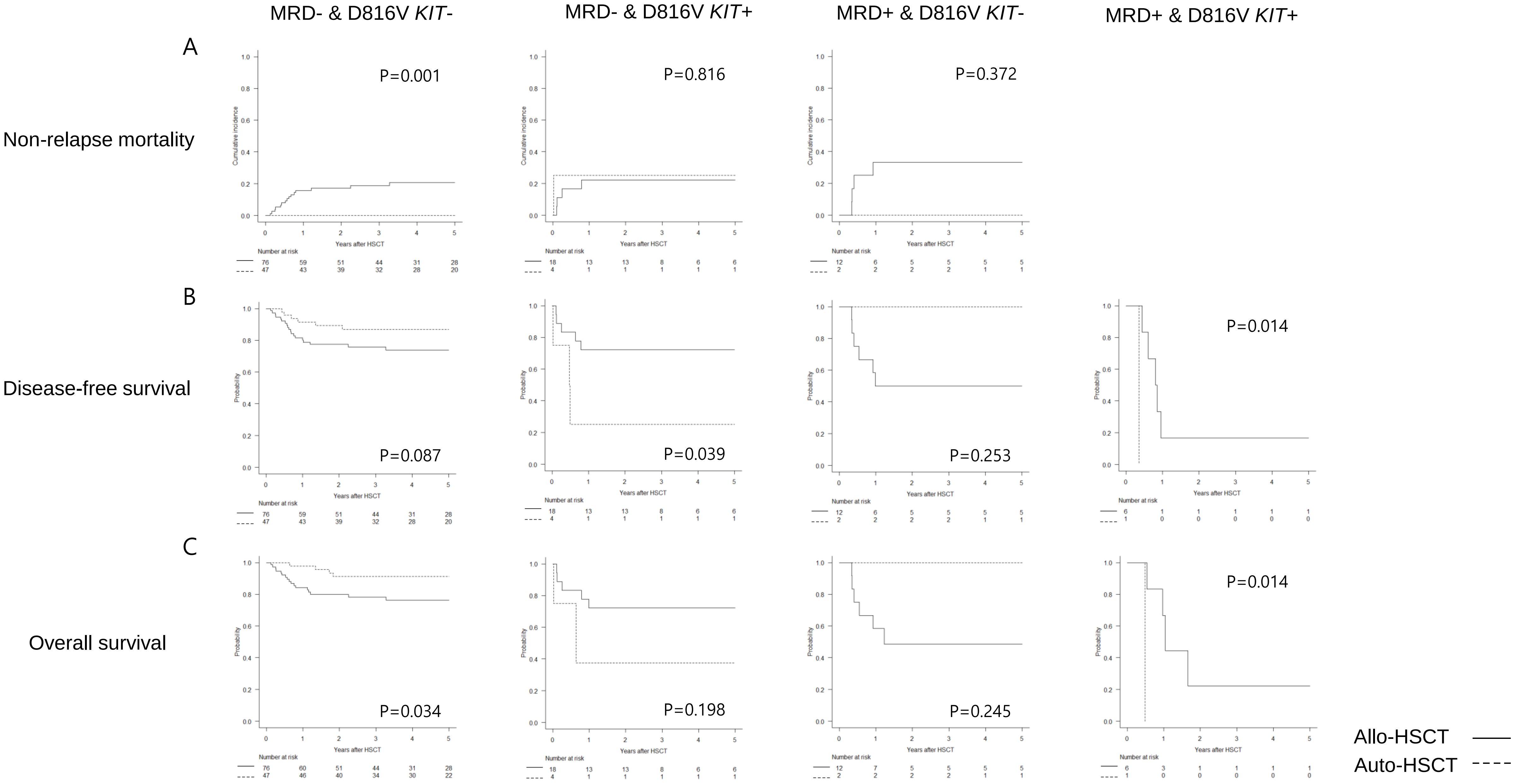

Supplement: Supplementary file 1 [file cancers-13-00336-s001.zip › 5-Supplementary Figures_Final_revised.pdf]
