# Supplementary material for: Prognostic Impacts of D816V KIT Mutation and Peri-Transplant RUNX1–RUNX1T1 MRD Monitoring on Acute Myeloid Leukemia with RUNX1–RUNX1T1
Source: Cancers (Basel). 2021 Jan 18;13(2):336. doi: 10.3390/cancers13020336 (PMC7831332; doi:10.3390/cancers13020336)
Supplement: Supplementary file 1 [file cancers-13-00336-s001.zip › cancers-1031767-supplementary matherials.docx]

Supplementary Material: Prognostic Impacts of D816V *KIT* Mutation and Peri-Transplant *RUNX1–RUNX1T1* MRD Monitoring on Acute Myeloid Leukemia with *RUNX1–RUNX1T1*

Byung-Sik Cho, Gi-June Min, Sung-Soo Park, Silvia Park, Young-Woo Jeon, Seung-Hwan Shin, Seung-Ah Yahng, Jae-Ho Yoon, Sung-Eun Lee, Ki-Seong Eom, Yoo-Jin Kim, Seok Lee, Chang-Ki Min, Seok-Goo Cho, Dong-Wook Kim, Jong Wook Lee, Myungshin Kim, Yonggoo Kim and Hee-Je Kim

Supplementary Methods: Quality control (QC) for *RUNX1-RUNX1T1* MRD assay

All procedures were performed according to the MIQE Guidelines: Minimum Information for Publication of Quantitative Real-Time PCR Experiments [1]. Detection and quantification of MRD using qRT-PCR were followed by previous studies [2–4]. Our laboratory had been certified as clinical laboratory for genetic testing by Korean Association of External Quality Assessment Service and Korean Institute of Genetic Testing Evaluation. Each organization performs proficiency testing of *RUNX1-RUNX1T1* twice per year and we have showed excellent results. Internal QC is carried out in every batch using adequate controls including negative and positive materials with known level of *RUNX1-RUNX1T1*. Reproducibility of the internal QC material was evaluated as within run and between run precision and defined acceptable when the coefficient of variation was less than 5%. Limit of detection was evaluated at the time of test setting and determined as 1 × 10^−5^. It is reevaluated when the lot number of reagent is changed.

1. Bustin, S.A.; Benes, V.; Garson, J.A.; Hellemans, J.; Huggett, J.; Kubista, M.; Mueller, R.; Nolan, T.; Pfaffl, M.W.; Shipley, G.L., et al. The MIQE guidelines: minimum information for publication of quantitative real-time PCR experiments. *Clin Chem* **2009**, *55*, 611–622, doi:10.1373/clinchem.2008.112797.

2. van der Velden, V.H.; Hochhaus, A.; Cazzaniga, G.; Szczepanski, T.; Gabert, J.; van Dongen, J.J. Detection of minimal residual disease in hematologic malignancies by real-time quantitative PCR: principles, approaches, and laboratory aspects. *Leukemia* **2003**, *17*, 1013–1034, doi:10.1038/sj.leu.2402922.

3. Gabert, J.; Beillard, E.; van der Velden, V.H.; Bi, W.; Grimwade, D.; Pallisgaard, N.; Barbany, G.; Cazzaniga, G.; Cayuela, J.M.; Cave, H., et al. Standardization and quality control studies of 'real-time' quantitative reverse transcriptase polymerase chain reaction of fusion gene transcripts for residual disease detection in leukemia - a Europe Against Cancer program. *Leukemia* **2003**, *17*, 2318–2357, doi:10.1038/sj.leu.2403135.

4. Gorello, P.; Cazzaniga, G.; Alberti, F.; Dell'Oro, M.G.; Gottardi, E.; Specchia, G.; Roti, G.; Rosati, R.; Martelli, M.F.; Diverio, D., et al. Quantitative assessment of minimal residual disease in acute myeloid leukemia carrying nucleophosmin (NPM1) gene mutations. *Leukemia* **2006**, *20*, 1103–1108, doi:10.1038/sj.leu.2404149.

**Table S1.** Patient-, disease-, and transplant-related characteristics according to transplant type.

| **Variables** | **Overall**  **(*n* = 166)** | **Allo-HSCT**  **(*n* = 112)** | **Auto-HSCT**  **(*n* = 54)** | ***p*** |
| --- | --- | --- | --- | --- |
| Age at transplantation, years, *n* (%) |  |  |  |  |
| Median (range) | 40 (18–69) | 43 (18–69) | 34 (18–64) | 0.007 |
| Sex, *n* (%) |  |  |  | 0.957 |
| Male | 105 (63) | 71 (63) | 34 (63) |  |
| Female | 61 (37) | 41 (37) | 20 (37) |  |
| AML type, *n* (%) |  |  |  | 0.175 |
| De novo | 161 (97) | 107 (96) | 54 (100) |  |
| Therapy-related | 5 (3) | 5 (4) | 0 |  |
| WBC count per liter at diagnosis, n (%) |  |  |  |  |
| Median (range) | 8.65 (0.53–100.91) | 10.75 (0.53–100.91) | 5.82 (1.33–68.7) | 0.009 |
| Additional cytogenetic abnormalities, n (%) |  |  |  |  |
| Del(9q) | 12 (7) | 7 (6) | 5 (9) | 0.529 |
| Trisomy 8 | 2 (1) | 1 (1) | 1 (2) | 0.546 |
| Loss of sex chromosome | 104 (63) | 72 (64) | 32 (59) | 0.608 |
| Del(7q) | 3 (2) | 1 (1) | 2 (4) | 0.247 |
| Complex karyotype | 9 (5) | 7 (6) | 2 (4) | 0.719 |
| *KIT* mutations *n* (%) | 70 (42) | 59 (53) | 11 (20) | <0.001 |
| Exon 17-D816V | 29 (18) | 24 (21) | 5 (9) | 0.079 |
| Exon 17-D816Y | 14 (8) | 11 (10) | 3 (6) | 0.552 |
| Exon 17-D816H | 19 (11) | 16 (14) | 3 (6) | 0.122 |
| Exon 17-N822K | 25 (15) | 22 (20) | 3 (6) | 0.020 |
| Exon 8 | 5 (3) | 4 (4) | 1 (2) | 1.000 |
| *FLT3* mutations, n (%) |  |  |  |  |
| *FLT3-ITD* | 9 (5) | 5 (5) | 4 (7) | 0.732 |
| *FLT3-TKD* | 3 (2) | 3 (3) | 0 | 0.479 |
| Missing data | 9 (5) | 6 (5) | 3 (6) |  |
| Disease status at HSCT, *n* (%) |  |  |  | 0.031 |
| CR1 | 156 (94) | 102 (91) | 54 (100) |  |
| CR2 | 10 (6) | 10 (9) | 0 |  |
| Donor type, *n* (%) |  |  |  | <0.001 |
| Matched sibling | 64 (39) | 64 (57) | 0 |  |
| Matched unrelated | 25 (15) | 25 (22) | 0 |  |
| Haploidentical | 23 (14) | 23 (21) | 0 |  |
| Autologous | 54 (33) | 0 | 54 (100) |  |
| Stem cell source, *n* (%) |  |  |  | <0.001 |
| Peripheral blood | 112 (67) | 86 (77) | 25 (46) |  |
| Bone marrow | 28 (17) | 26 (23) | 2 (4) |  |
| Peripheral blood and bone marrow | 27 (16) | 0 | 27 (50) |  |
| Conditioning intensity, *n* (%) |  |  |  | <0.001 |
| Myeloablative | 105 (63) | 51 (46) | 54 (100) |  |
| Reduced-intensity | 61 (37) | 61 (54) | 0 |  |
| Interval from diagnosis to transplant, days |  |  |  |  |
| Median (range) | 194 (96–260) | 175 (96–260) | 202 (151–253) | 0.001 |
| CD34^+^ cells × 10^6^/kg in graft |  |  |  |  |
| Median (range) | 3.88 (0.73–16.73) | 4.86 (0.73–16.73) | 2.78 (1.01–7.24) | <0.001 |

Abbreviations: AML, acute myeloid leukemia; Allo-HSCT, allogeneic HSCT; Auto-HSCT, autologous HSCT; CR1, first complete remission; CR2, second complete remission; HSCT, hematopoietic stem cell transplantation; *n*, number; WBC, white blood cells.

**Table S2.** Factors affecting survival outcomes (Univariate analysis).

| **Univariate Variables** | **n** | **Cumulative Incidence of Relapse** | | **Cumulative Incidence of Non-Relapse Mortality** | | **Disease-Free Survival** | | **Overall Survival** | |
| --- | --- | --- | --- | --- | --- | --- | --- | --- | --- |
|  |  | **HR (95% CI)** | ***P* Value** | **HR (95% CI)** | ***P* Value** | **HR (95% CI)** | ***P* Value** | **HR (95% CI)** | ***P* Value** |
| *KIT* mutations  *KIT* unmutated  *KIT* mutated | 96  70 | 1  2.28 (0.96–5.41) | 0.119 | 1  2.66 (1.16–6.09) | 0.020 | 1  2.47 (1.36–4.49) | 0.003 | 1  2.99 (1.55–5.75) | 0.001 |
| D816V *KIT* mutation  D816V *KIT* unmutated  D816V *KIT* mutated | 138  28 | 4.68 (1.97–11.1) | <0.001 | 1  1.52 (0.57–4.07) | 0.408 | 1  2.70 (1.44–5.08) | 0.002 | 1  2.59 (1.31–5.11) | 0.006 |
| D816V *KIT* mutation  No *KIT* mutations  D816V *KIT* mutated  Other *KIT* mutations | 96  28  42 | 1  4.57 (1.81–11.52)  0.92 (0.25–3.37) | 0.004  0.001  0.890 | 1  1.86 (0.57–6.030  3.16 (1.31–7.62) | 0.038  0.304  0.011 | 1  3.14 (1.54–6.42)  2.06 (1.03–4.15) | 0.005  0.002  0.042 | 1  3.36 (1.53–7.42)  2.74 (1.31–5.76) | 0.004  0.003  0.008 |
| *RUNX1-RUNX1T1* levels at pre-HSCT  ≥ 3 log reduction  < 3 log reduction | 145  21 | 1  5.80 (2.39–14.03) | <0.001 | 1  1.59 (0.54–4.67) | 0.538 | 1  3.13 (1.61–6.7) | <0.001 | 1  3.38 (1.68–6.82) | <0.001 |
| *RUNX1-RUNX1T1* levels at 1 month after HSCT  ≥ 3 log reduction  < 3 log reduction | 79  4 | 1  11.1 (2.96–41.66) | <0.001 | 1  0.05 (0–11499.6) | 0.378 | 1  3.81 (1.13–12.8) | 0.023 | 1  4.58 (1.33–15.7) | 0.008 |
| *RUNX1-RUNX1T1* levels at 3 months after HSCT  ≥ 3 log reduction  < 3 log reduction | 96  6 | 1  29.8 (8.71–102.0) | <0.001 | 0.05 (0–42854.4) | 0.389 | 1  8.62 (3.12–23.85) | <0.001 | 1  6.07 (2.18–20.64) | <0.001 |
| Additional cytogenetic abnormalities  Del(9q)  Trisomy 8  Loss of sex chromosome  Del(7q)  Complex karyotype | 12  2  104  3  9 | 1.52 (0.354–6.53)  3.67 (0.49–27.4)  0.44 (0.19–1.04)  0.05 (0–21506.8)  0.05 (0–111.8) | 0.574  0.204  0.061  0.648  0.437 | 2.90 (0.99–8.51)  0.05 (0–397333.8)  1.76 (0.70–4.43)  0.05 (0–12299.2)  0.69 (0.09–5.08) | 0.053  0.710  0.232  0.633  0.713 | 2.22 (0.94–5.26)  1.71 (0.24–12.44)  0.88 (0.48–1.59)  0.05 (0–388.2)  0.35 (0.05–2.54) | 0.069  0.595  0.667  0.509  0.299 | 2.23 (0.87–5.70)  2.12 (0.29–15.41)  0.94 (0.49–1.79)  0.05 (0–759.3)  0.41 (0.06–3.00) | 0.095  0.460  0.847  0.538  0.379 |
| Age | 166 | 1.01 (0.98–1.04) | 0.658 | 1.03 (1.002–1.07) | 0.039 | 1.02 (0.998–1.05) | 0.068 | 1.03 (1.01–1.06) | 0.012 |
| Sex  Male  Female | 105  61 | 1  1.21 (0.49–2.99) | 0.686 | 1  0.68 (0.28–1.64) | 0.388 | 1  0.75 (0.40–1.40) | 0.364 | 1  0.75 (0.38–1.47) | 0.399 |
| WBC at diagnosis | 166 | 1.00 (1.00–1.00) | 0.588 | 1.00 (1.00–1.00) | 0.361 | 1.00 (1.00–1.00) | 0.764 | 1.00 (1.00–1.00) | 0.738 |
| AML type  De novo  Therapy-related | 161  5 | 1  0.05 (0–4061.0) | 0.599 | 1  1.43 (0.19–10.59) | 0.727 | 1  0.75 (0.10–5.47) | 0.779 | 1  0.97 (1.33–7.09) | 0.978 |
| Disease state  CR1  CR2 | 156  10 | 1  4.23 (1.24–14.39) | 0.024 | 1  2.97 (0.88–10.0) | 0.079 | 1  3.50 (1.48–8.29) | 0.004 | 1  3.23 (1.26–8.30) | 0.015 |
| Transplant type  Allo-HSCT  Auto-HSCT | 112  54 | 1  1.4 (0.59–3.32) | 0.446 | 1  0.08 (0.01–0.62) | 0.015 | 1  0.54 (0.27–1.10) | 0.089 | 1  0.40 (0.18–0.91) | 0.028 |
| Stem cell source  PB  BM  PB+BM | 111  28  27 | 1  1.50 (0.53–4.21)  0.84 (0.24–2.94) | 0.668  0.442  0.783 | 1  0.58 (0.17–1.94)  0.19 (0.03–1.42) | 0.202  0.375  0.106 | 1  0.94 (0.43–2.03)  0.45 (016–1.28) | 0.324  0.872  0.134 | 1  0.71 (0.30–1.69)  0.12 (0.02–0.84) | 0.085  0.434  0.033 |
| Conditioning intensity  Myeloablative  Reduced-intensity | 105  61 | 1  0.98 (0.409–2.41) | 0.971 | 1  3.1 (1.35–7.0) | 0.008 | 1  1.82 (1.01–3.26) | 0.045 | 1  2.04 (1.09–3.82) | 0.026 |
| Acute GVHD*  < Grade II  Grade II  Grade III–IV | 65  34  13 | 1  1.72 (0.52–5.62)  1.71 (0.21–14.3) | 0.647  0.373  0.619 | 1  1.50 (0.52–4.32)  8.80 (3.35–23.16) | <0.001  0.454  <0.001 | 1  1.57 (0.72–3.49)  6.09 (2.68–13.85) | <0.001  0.253  <0.001 | 1  1.38 (0.59–3.23)  6.70 (2.90–15.46) | <0.001  0.458  <0.001 |
| Chronic GVHD*  None or mild  Moderate and severe | 74  38 | 1  0.61 (0.17–2.26) | 0.461 | 1  0.99 (0.42–2.33) | 0.977 | 1  0.85 (0.42–1.73) | 0.647 | 1  0.84 (0.40–1.78) | 0.650 |

Abbreviations: acute myeloid leukemia; Allo-HSCT, allogeneic HSCT; Auto-HSCT, autologous HSCT; BM, bone marrow; CI, confidence interval; CR1, first complete remission; CR2, second complete remission; GVHD, graft-versus-host disease; HSCT, hematopoietic stem cell transplantation; *n*, number; PB, peripheral blood; WBC, white blood cells. *GVHD was classified as the revised NIH consensus criteria (*Biol Blood Marrow Transplant* **2015**; *21*; 389–401).

**Table S3.** Multivariate analysis to reveal an impact of *KIT* or D816 *KIT* mutations on survival outcomes

| **Model #1** | n | **Relapse** | | **Non-Relapse Mortality** | | **Disease-Free Survival** | | **Overall Survival** | | |
| --- | --- | --- | --- | --- | --- | --- | --- | --- | --- | --- |
|  |  | **HR (95% CI)** | ***P* Value** | **HR (95% CI)** | ***P* Value** | **HR (95% CI)** | ***P* Value** | | **HR (95% CI)** | ***P* Value** |
| *KIT* mutations |  |  |  |  |  |  |  | |  |  |
| Unmutated | 96 | 1 |  | 1 |  | 1 |  | | 1 |  |
| Mutated | 70 | 3.08 (1.18–7.0) | 0.021 | 1.77 (0.76–4.09) | 0.184 | 3.05 (1.54–6.04) | 0.001 | | 3.33 (1.58–7.02) | 0.002 |
| Age at HSCT, years | 166 |  |  | 1.02 (0.99–1.06) | 0.164 | 1.02 (0.99–1.04) | 0.157 | | 1.03 (1.00–1.05) | 0.046 |
| Disease state |  |  |  |  |  |  |  | |  |  |
| CR1 | 75 | 1 |  |  |  | 1 |  | | 1 |  |
| CR2 | 79 | 7.28 (1.88–28.1) | 0.004 |  |  | 5.33 (2.0–14.13) | 0.001 | | 4.64 (1.61–13.32) | 0.004 |
| Transplant type |  |  |  |  |  |  |  | |  |  |
| Allo-HSCT | 112 |  |  | 1 |  | 1 |  | | 1 |  |
| Auto-HSCT | 54 |  |  | 0.12 (0.16–0.92) | 0.041 | 1.08 (0.49–2.38) | 0.849 | | 0.84 (0.34–2.05) | 0.699 |
| **Model #2** | **n** | **Relapse** | | **Non-Relapse Mortality** | | **Disease-Free Survival** | | | **Overall Survival** | |
|  |  | **HR (95% CI)** | ***P* Value** | **HR (95% CI)** | ***P* Value** | **HR (95% CI)** | ***P* Value** | | **HR (95% CI)** | ***P* Value** |
| D816V *KIT* mutation |  |  |  |  |  |  |  | |  |  |
| Unmutated | 145 | 1 |  |  |  | 1 |  | | 1 |  |
| Mutated | 21 | 5.60 (2.27–13.8) | <0.001 |  |  | 2.95 (1.49–5.80) | 0.002 | | 2.58 (1.25–5.31) | 0.010 |
| Age at HSCT, years | 166 |  |  | 1.03 (0.99–1.06) | 0.158 | 1.02 (0.99–1.04) | 0.106 | | 1.03 (1.00–1.05) | 0.031 |
| Disease state |  |  |  |  |  |  |  | |  |  |
| CR1 | 75 | 1 |  |  |  | 1 |  | | 1 |  |
| CR2 | 79 | 6.41 (1.79–23.0) | 0.004 |  |  | 3.85 (1.54–9.61) | 0.004 | | 3.12 (1.16–8.40) | 0.024 |
| Transplant type |  |  |  |  |  |  |  | |  |  |
| Allo-HSCT | 112 |  |  | 1 |  | 1 |  | | 1 |  |
| Auto-HSCT | 54 |  |  | 0.10 (0.01–0.73) | 0.023 | 0.90 (0.42–1.94) | 0.788 | | 0.65 (0.27–1.56) | 0.336 |

Abbreviations: Allo-HSCT, allogeneic HSCT; Auto-HSCT, autologous HSCT; CI, confidence interval; CR1, first complete remission; CR2, second complete remission; HR, Hazard ratio; HSCT, hematopoietic stem cell transplantation; *n*, number.

**Table S4.** Impact of transplant type on kinetics of *RUNX1-RUNX1T1* transcript levels*.

| **Variables** | ***n*** | **Time Range of**  **the Determinations** | **Non-Evaluable Patients** | **Log_10_ Transformed Transcript Levels** | | | **Log Reduction** | | |
| --- | --- | --- | --- | --- | --- | --- | --- | --- | --- |
|  |  |  |  | **Allo-HSCT** | **Auto-HSCT** | ***p*** | **Allo-HSCT** | **Auto-HSCT** | ***p*** |
| Pre-HSCT | 166 | Between days 47 and 14 before HSCT | NA | −3.5 ± 0.14 | −4.1 ± 0.19 | 0.023 | −4.20 ± 0.14 | −4.78 ± 0.18 | 0.015 |
| 1 month  after HSCT | 83 | Between days 25 and 35 after HSCT | NRM < 1 month (*n* = 1)  Relapse <1 month (*n* = 0)  MRD not performed (*n* = 82) | −4.24 ± 0.17 | −5.13 ± 0.39 | 0.046 | −4.87 ± 0.17 | −5.88 ± 0.37 | 0.024 |
| 3 months  after HSCT | 102 | Between days 84 and 99 after HSCT | NRM < 3 months (*n* = 5)  Relapse <3 months (*n* = 0)  MRD not performed (*n* = 59) | −4.84 ± 0.15 | −5.03 ± 0.37 | 0.636 | −5.50 ± 0.15 | −5.76 ± 0.37 | 0.504 |

Abbreviations: HSCT, hematopoietic stem cell transplantation; *n*, number; NA, non-applicable; NRM, non-relapse mortality; MRD, measurable residual disease. **RUNX1-RUNX1T1* transcript levels were normalized with respect to the number of *ABL1* transcripts and expressed as copy numbers per 10^5^ copies of *ABL1*. Data were expressed as mean ± SEM.

**Table S5.** Sensitivity and specificity of *RUNX1-RUNX1T1* MRD positive patients defined by various cutoffs at each time points*.

| **Various Cutoffs at Each Time Point**  **of MRD Assessment** | **MRD Positive Patents (%)** | **Relapsed Patients Among MRD Positive Patients** | **Sensitivity** | **MRD Negative Patents (%)** | **Non-Relapsed Patients Among MRD Negative Patients** | **Specificity** |
| --- | --- | --- | --- | --- | --- | --- |
| Pre-HSCT (*n* = 166) |  |  |  |  |  |  |
| 1000 copies | 15 (9) | 5 | 33% | 151 (91) | 135 | 89% |
| 500 copies | 21 (13) | 7 | 33% | 145 (87) | 131 | 90% |
| 250 copies | 31 (19) | 8 | 26% | 135 (81) | 122 | 90% |
| 100 copies | 53 (32) | 11 | 21% | 113 (68) | 103 | 91% |
| 50 copies | 73 (44) | 16 | 22% | 93 (56) | 88 | 95% |
| 10 copies | 111 (67) | 18 | 16% | 55 (33) | 52 | 95% |
| 0 copies | 130 (78) | 21 | 16% | 36 (22) | 36 | 100% |
| 3 log reduction | 21 (13) | 8 | 38% | 145 (87) | 132 | 91% |
| 4 log reduction | 71 (43) | 15 | 21% | 95 (57%) | 89 | 94% |
| 1 month after HSCT (*n* = 83) |  |  |  |  |  |  |
| 1000 copies | 3 (4) | 2 | 67% | 80 (96) | 70 | 88% |
| 500 copies | 4 (5) | 2 | 50% | 79 (95) | 69 | 87% |
| 250 copies | 7 (8) | 3 | 43% | 76 (92) | 67 | 88% |
| 100 copies | 20 (24) | 7 | 35% | 63 (76) | 58 | 92% |
| 50 copies | 28 (34) | 8 | 29% | 55 (66) | 51 | 93% |
| 10 copies | 43 (52) | 10 | 23% | 40 (48) | 38 | 95% |
| 0 copies | 50 (60) | 11 | 22% | 33 (40) | 32 | 97% |
| 3 log reduction | 4 (5) | 3 | 75% | 79 (95) | 70 | 89% |
| 4 log reduction | 27 (33) | 8 | 30% | 56 (68) | 52 | 93% |
| 3 months after HSCT (*n* = 102) |  |  |  |  |  |  |
| 1000 copies | 4 (4) | 3 | 75% | 98 (96) | 90 | 92% |
| 500 copies | 5 (5) | 4 | 80% | 97 (95) | 90 | 93% |
| 250 copies | 8 (8) | 6 | 75% | 94 (92) | 89 | 95% |
| 100 copies | 13 (13) | 6 | 46% | 89 (87) | 84 | 94% |
| 50 copies | 17 (17) | 7 | 41% | 85 (83) | 81 | 95% |
| 10 copies | 30 (29) | 9 | 30% | 72 (71) | 70 | 97% |
| 0 copies | 45 (44) | 10 | 22% | 57 (56) | 56 | 98% |
| 3 log reduction | 6 (6) | 5 | 83% | 96 (94) | 90 | 94% |
| 4 log reduction | 14 (14) | 7 | 50% | 88 (86) | 84 | 96% |

Abbreviations: HSCT, hematopoietic stem cell transplantation; n, number: MRD, measurable residual disease. **RUNX1-RUNX1T1* transcript levels were normalized with respect to the number of *ABL1* transcripts and expressed as copy numbers per 10^5^ copies of *ABL1*.

**Table S6.** Multivariate analysis to reveal an impact of *RUNX1-RUNX1T1* quantification at each time point on survival outcomes.

| **Model #1 (*n* = 166)** | **n** | **Relapse** | | **Disease-Free Survival** | | **Overall Survival** | | |
| --- | --- | --- | --- | --- | --- | --- | --- | --- |
|  |  | **HR (95% CI)** | ***P* Value** | **HR (95% CI)** | ***P* Value** | | **HR (95% CI)** | ***P* Value** |
| ***RUNX1-RUNX1T1* Levels at Pre-HSCT** |  |  |  |  |  | |  |  |
| ≥ 3 log reduction | 145 | 1 |  | 1 |  | | 1 |  |
| < 3 log reduction | 21 | 6.62 (2.67–16.39) | <0.001 | 2.92 (1.48–5.75) | 0.002 | | 2.97 (1.45–6.08) | 0.003 |
| Age at HSCT, years | 166 |  |  | 1.02 (0.99–1.04) | 0.174 | | 1.03 (1.00–1.05) | 0.048 |
| Disease state |  |  |  |  |  | |  |  |
| CR1 | 156 | 1 |  | 1 |  | | 1 |  |
| CR2 | 10 | 5.76 (1.63–20.39) | 0.007 | 3.12 (1.28–7.59) | 0.012 | | 2.60 (0.99–6.82) | 0.052 |
| Transplant type |  |  |  |  |  | |  |  |
| Allo-HSCT | 112 |  |  | 1 |  | | 1 |  |
| Auto-HSCT | 54 |  |  | 0.79 (0.37–1.67) | 0.535 | | 0.60 (0.25–1.41) | 0.241 |
| **Model #2 (*n* = 83)** | ***n*** | **Relapse** | | **Disease-Free Survival** | | | **Overall Survival** | |
|  |  | **HR (95% CI)** | ***P* Value** | **HR (95% CI)** | ***P* Value** | | **HR (95% CI)** | ***P* Value** |
| *RUNX1-RUNX1T1* levels at 1 month after HSCT |  |  |  |  |  | |  |  |
| ≥ 3 log reduction | 79 | 1 |  | 1 |  | | 1 |  |
| < 3 log reduction | 4 | 13.44 (3.39–53.38) | <0.001 | 3.33 (0.94–11.81) | 0.062 | | 4.33 (1.22–15.40) | 0.023 |
| Age at HSCT, years | 83 |  |  | 1.00 (0.98–1.03) | 0.764 | | 1.02 (0.99–1.05) | 0.249 |
| Disease state |  |  |  |  |  | |  |  |
| CR1 | 75 | 1 |  | 1 |  | | 1 |  |
| CR2 | 8 | 6.20 (1.59–24.2) | 0.009 | 2.92 (1.08–7.91) | 0.035 | | 2.35 (0.79–7.04) | 0.127 |
| Transplant type |  |  |  |  |  | |  |  |
| Allo-HSCT | 70 |  |  | 1 |  | | 1 |  |
| Auto-HSCT | 13 |  |  | 0.24 (0.03–1.80) | 0.164 | | 0 (0–3.165E+244) | 0.965 |
| **Model #3 (*n* = 102)** |  | **Relapse** | | **Disease-Free Survival** | | | **Overall Survival** | |
|  |  | **HR (95% CI)** | ***P* Value** | **HR (95% CI)** | ***P* Value** | | **HR (95% CI)** | ***P* Value** |
| *RUNX1-RUNX1T1* levels at 3 months after HSCT |  |  |  |  |  | |  |  |
| ≥ 3 log reduction | 96 | 1 |  | 1 |  | | 1 |  |
| < 3 log reduction | 6 | 36.36 (9.91–133.37) | <0.001 | 6.49 (2.14–19.75) | 0.001 | | 4.69 (1.40–15.71) | 0.012 |
| Age at HSCT, years | 102 |  |  | 1.03 (0.99–1.06) | 0.152 | | 1.03 (0.99–1.07) | 0.074 |
| Disease state |  |  |  |  |  | |  |  |
| CR1 | 94 | 1 |  | 1 |  | | 1 |  |
| CR2 | 8 | 6.02 (1.15–31.45) | 0.033 | 3.53 (1.17–10.65) | 0.025 | | 2.67 (0.77–9.29) | 0.123 |
| Transplant type |  |  |  |  |  | |  |  |
| Allo-HSCT | 86 |  |  | 1 |  | | 1 |  |
| Auto-HSCT | 16 |  |  | 0.60 (0.14–2.63) | 0.502 | | 0.56 (0.13–2.46) | 0.446 |

Abbreviations: Allo-HSCT, allogeneic HSCT; Auto-HSCT, autologous HSCT; CI, confidence interval; CR1, first complete remission; CR2, second complete remission; HSCT, hematopoietic stem cell transplantation; *n*, number.
