# Supplementary material for: Prognostic Impacts of D816V KIT Mutation and Peri-Transplant RUNX1–RUNX1T1 MRD Monitoring on Acute Myeloid Leukemia with RUNX1–RUNX1T1
Source: Cancers (Basel). 2021 Jan 18;13(2):336. doi: 10.3390/cancers13020336 (PMC7831332; doi:10.3390/cancers13020336)
Supplement: Supplementary file 1 [file cancers-13-00336-s001.zip › cancers-1031767-supplementary matherials.pdf]

Supplementary Material: Prognostic Impacts of D816V *KIT* Mutation and Peri-Transplant *RUNX1–RUNX1T1* MRD Monitoring on Acute Myeloid Leukemia with *RUNX1–RUNX1T1*

Byung-Sik Cho, Gi-June Min, Sung-Soo Park, Silvia Park, Young-Woo Jeon, Seung-Hwan Shin, Seung-Ah Yahng, Jae-Ho Yoon, Sung-Eun Lee, Ki-Seong Eom, Yoo-Jin Kim, Seok Lee, Chang-Ki Min, Seok-Goo Cho, Dong-Wook Kim, Jong Wook Lee, Myungshin Kim, Yonggoo Kim and Hee-Je Kim

Supplementary Methods: Quality control (QC) for *RUNX1-RUNX1T1* MRD assay

All procedures were performed according to the MIQE Guidelines: Minimum Information for Publication of Quantitative Real-Time PCR Experiments [1]. Detection and quantification of MRD using qRT-PCR were followed by previous studies [2–4]. Our laboratory had been certified as clinical laboratory for genetic testing by Korean Association of External Quality Assessment Service and Korean Institute of Genetic Testing Evaluation. Each organization performs proficiency testing of *RUNX1-RUNX1T1* twice per year and we have showed excellent results. Internal QC is carried out in every batch using adequate controls including negative and positive materials with known level of *RUNX1-RUNX1T1*. Reproducibility of the internal QC material was evaluated as within run and between run precision and defined acceptable when the coefficient of variation was less than 5%. Limit of detection was evaluated at the time of test setting and determined as 1×10^{-5}. It is reevaluated when the lot number of reagent is changed.

1. Bustin, S.A.; Benes, V.; Garson, J.A.; Hellemans, J.; Huggett, J.; Kubista, M.; Mueller, R.; Nolan, T.; Pfaffl, M.W.; Shipley, G.L., et al. The MIQE guidelines: minimum information for publication of quantitative real-time PCR experiments. *Clin Chem* **2009**, *55*, 611–622, doi:10.1373/clinchem.2008.112797.
2. van der Velden, V.H.; Hochhaus, A.; Cazzaniga, G.; Szczepanski, T.; Gabert, J.; van Dongen, J.J. Detection of minimal residual disease in hematologic malignancies by real-time quantitative PCR: principles, approaches, and laboratory aspects. *Leukemia* **2003**, *17*, 1013–1034, doi:10.1038/sj.leu.2402922.
3. Gabert, J.; Beillard, E.; van der Velden, V.H.; Bi, W.; Grimwade, D.; Pallisgaard, N.; Barbany, G.; Cazzaniga, G.; Cayuela, J.M.; Cave, H., et al. Standardization and quality control studies of 'real-time' quantitative reverse transcriptase polymerase chain reaction of fusion gene transcripts for residual disease detection in leukemia - a Europe Against Cancer program. *Leukemia* **2003**, *17*, 2318–2357, doi:10.1038/sj.leu.2403135.
4. Gorello, P.; Cazzaniga, G.; Alberti, F.; Dell'Oro, M.G.; Gottardi, E.; Specchia, G.; Roti, G.; Rosati, R.; Martelli, M.F.; Diverio, D., et al. Quantitative assessment of minimal residual disease in acute myeloid leukemia carrying nucleophosmin (NPM1) gene mutations. *Leukemia* **2006**, *20*, 1103–1108, doi:10.1038/sj.leu.2404149.

Table S1. Patient-, disease-, and transplant-related characteristics according to transplant type.

Variables	Overall (*n* = 166)	Allo-HSCT (*n* = 112)	Auto-HSCT (*n* = 54)	*p*
Age at transplantation, years, *n* (%)				
Median (range)	40 (18–69)	43 (18–69)	34 (18–64)	0.007
Sex, *n* (%)				0.957
Male	105 (63)	71 (63)	34 (63)	
Female	61 (37)	41 (37)	20 (37)	
AML type, *n* (%)				0.175
De novo	161 (97)	107 (96)	54 (100)	
Therapy-related	5 (3)	5 (4)	0	
WBC count per liter at diagnosis, n (%)				
Median (range)	8.65 (0.53–100.91)	10.75 (0.53–100.91)	5.82 (1.33–68.7)	0.009
Additional cytogenetic abnormalities, n (%)				
Del(9q)	12 (7)	7 (6)	5 (9)	0.529

Trisomy 8	2 (1)	1 (1)	1 (2)	0.546
Loss of sex chromosome	104 (63)	72 (64)	32 (59)	0.608
Del(7q)	3 (2)	1 (1)	2 (4)	0.247
Complex karyotype	9 (5)	7 (6)	2 (4)	0.719
KIT mutations *n* (%)	70 (42)	59 (53)	11 (20)	<0.001
Exon 17-D816V	29 (18)	24 (21)	5 (9)	0.079
Exon 17-D816Y	14 (8)	11 (10)	3 (6)	0.552
Exon 17-D816H	19 (11)	16 (14)	3 (6)	0.122
Exon 17-N822K	25 (15)	22 (20)	3 (6)	0.020
Exon 8	5 (3)	4 (4)	1 (2)	1.000
FLT3 mutations, n (%)				
FLT3-ITD	9 (5)	5 (5)	4 (7)	0.732
FLT3-TKD	3 (2)	3 (3)	0	0.479
Missing data	9 (5)	6 (5)	3 (6)	
Disease status at HSCT, *n* (%)				0.031
CR1	156 (94)	102 (91)	54 (100)	
CR2	10 (6)	10 (9)	0	
Donor type, *n* (%)				<0.001
Matched sibling	64 (39)	64 (57)	0	
Matched unrelated	25 (15)	25 (22)	0	
Haploidentical	23 (14)	23 (21)	0	
Autologous	54 (33)	0	54 (100)	
Stem cell source, *n* (%)				<0.001
Peripheral blood	112 (67)	86 (77)	25 (46)	
Bone marrow	28 (17)	26 (23)	2 (4)	
Peripheral blood and bone marrow	27 (16)	0	27 (50)	
Conditioning intensity, *n* (%)				<0.001
Myeloablative	105 (63)	51 (46)	54 (100)	
Reduced-intensity	61 (37)	61 (54)	0	
Interval from diagnosis to transplant, days				
Median (range)	194 (96–260)	175 (96–260)	202 (151–253)	0.001
$CD34^+$ cells × 10^6/kg in graft				
Median (range)	3.88 (0.73–16.73)	4.86 (0.73–16.73)	2.78 (1.01–7.24)	<0.001

Abbreviations: AML, acute myeloid leukemia; Allo-HSCT, allogeneic HSCT; Auto-HSCT, autologous HSCT; CR1, first complete remission; CR2, second complete remission; HSCT, hematopoietic stem cell transplantation; *n*, number; WBC, white blood cells.

Table S2. Factors affecting survival outcomes (Univariate analysis).

Univariate Variables	n	Cumulative Incidence of Relapse		Cumulative Incidence of Non-Relapse Mortality		Disease-Free Survival		Overall Survival	
		HR (95% CI)	*P* Value	HR (95% CI)	*P* Value	HR (95% CI)	*P* Value	HR (95% CI)	*P* Value
KIT mutations									
KIT unmutated	96	1		1		1		1	
KIT mutated	70	2.28 (0.96–5.41)	0.119	2.66 (1.16–6.09)	0.020	2.47 (1.36–4.49)	0.003	2.99 (1.55–5.75)	0.001
D816V *KIT* mutation									
D816V *KIT* unmutated	138			1		1		1	
D816V *KIT* mutated	28	4.68 (1.97–11.1)	<0.001	1.52 (0.57–4.07)	0.408	2.70 (1.44–5.08)	0.002	2.59 (1.31–5.11)	0.006
D816V *KIT* mutation		1	0.004	1	0.038	1	0.005	1	0.004
No *KIT* mutations	96	4.57 (1.81–		1.86 (0.57–		3.14 (1.54–		3.36 (1.53–	
D816V *KIT* mutated	28	11.52)	0.001	6.030	0.304	6.42)	0.002	7.42)	0.003
Other *KIT* mutations	42	0.92 (0.25–	0.890	3.16 (1.31–	0.011	2.06 (1.03–	0.042	2.74 (1.31–	0.008
		3.37)		7.62)		4.15)		5.76)	
RUNX1-RUNX1T1 levels at pre-HSCT									
≥ 3 log reduction	145	1		1		1		1	
< 3 log reduction	21	5.80 (2.39–14.03)	<0.001	1.59 (0.54–4.67)	0.538	3.13 (1.61–6.7)	<0.001	3.38 (1.68–6.82)	<0.001
RUNX1-RUNX1T1 levels at 1 month after HSCT									
≥ 3 log reduction	79	1		1		1		1	
< 3 log reduction	4	11.1 (2.96–41.66)	<0.001	0.05 (0–11499.6)	0.378	3.81 (1.13–12.8)	0.023	4.58 (1.33–15.7)	0.008
RUNX1-RUNX1T1 levels at 3 months after HSCT									
≥ 3 log reduction	96	1				1		1	
< 3 log reduction	6	29.8 (8.71–102.0)	<0.001	0.05 (0–42854.4)	0.389	8.62 (3.12–23.85)	<0.001	6.07 (2.18–20.64)	<0.001

Additional cytogenetic abnormalities									
Del(9q)	12	1.52 (0.354–6.53)	0.574	2.90 (0.99–8.51)	0.053	2.22 (0.94–5.26)	0.069	2.23 (0.87–5.70)	0.095
Trisomy 8	2	3.67 (0.49–27.4)	0.204	0.05 (0–397333.8)	0.710	1.71 (0.24–12.44)	0.595	2.12 (0.29–15.41)	0.460
Loss of sex chromosome	104	0.44 (0.19–1.04)	0.061	1.76 (0.70–4.43)	0.232	0.88 (0.48–1.59)	0.667	0.94 (0.49–1.79)	0.847
Del(7q)	3	0.05 (0–21506.8)	0.648	0.05 (0–12299.2)	0.633	0.05 (0–388.2)	0.509	0.05 (0–759.3)	0.538
Complex karyotype	9	0.05 (0–111.8)	0.437	0.69 (0.09–5.08)	0.713	0.35 (0.05–2.54)	0.299	0.41 (0.06–3.00)	0.379
Age	166	1.01 (0.98–1.04)	0.658	1.03 (1.002–1.07)	0.039	1.02 (0.998–1.05)	0.068	1.03 (1.01–1.06)	0.012
Sex									
Male	105	1		1		1		1	
Female	61	1.21 (0.49–2.99)	0.686	0.68 (0.28–1.64)	0.388	0.75 (0.40–1.40)	0.364	0.75 (0.38–1.47)	0.399
WBC at diagnosis	166	1.00 (1.00–1.00)	0.588	1.00 (1.00–1.00)	0.361	1.00 (1.00–1.00)	0.764	1.00 (1.00–1.00)	0.738
AML type									
De novo	161	1		1		1		1	
Therapy-related	5	0.05 (0–4061.0)	0.599	1.43 (0.19–10.59)	0.727	0.75 (0.10–5.47)	0.779	0.97 (1.33–7.09)	0.978
Disease state									
CR1	156	1		1		1		1	
CR2	10	4.23 (1.24–14.39)	0.024	2.97 (0.88–10.0)	0.079	3.50 (1.48–8.29)	0.004	3.23 (1.26–8.30)	0.015
Transplant type									
Allo-HSCT	112	1		1		1		1	
Auto-HSCT	54	1.4 (0.59–3.32)	0.446	0.08 (0.01–0.62)	0.015	0.54 (0.27–1.10)	0.089	0.40 (0.18–0.91)	0.028
Stem cell source			0.668		0.202		0.324		0.085
PB	111	1		1		1		1	
BM	28		0.442		0.375		0.872		0.434

PB+BM	27	1.50 (0.53–4.21)	0.783	0.58 (0.17–1.94)	0.106	0.94 (0.43–2.03)	0.134	0.71 (0.30–1.69)	0.033
		0.84 (0.24–2.94)		0.19 (0.03–1.42)		0.45 (016–1.28)		0.12 (0.02–0.84)	
Conditioning intensity									
Myeloablative	105	1		1		1		1	
Reduced-intensity	61	0.98 (0.409–2.41)	0.971	3.1 (1.35–7.0)	0.008	1.82 (1.01–3.26)	0.045	2.04 (1.09–3.82)	0.026
Acute GVHD*		1	0.647	1	<0.001	1	<0.001	1	<0.001
< Grade II	65	1.72 (0.52–5.62)	0.373	1.50 (0.52–4.32)	0.454	1.57 (0.72–3.49)	0.253	1.38 (0.59–3.23)	0.458
Grade II	34								
Grade III–IV	13	1.71 (0.21–14.3)	0.619	8.80 (3.35–23.16)	<0.001	6.09 (2.68–13.85)	<0.001	6.70 (2.90–15.46)	<0.001
Chronic GVHD*			0.461		0.977		0.647		0.650
None or mild	74	1		1		1		1	
Moderate and severe	38	0.61 (0.17–2.26)		0.99 (0.42–2.33)		0.85 (0.42–1.73)		0.84 (0.40–1.78)	

Abbreviations: acute myeloid leukemia; Allo-HSCT, allogeneic HSCT; Auto-HSCT, autologous HSCT; BM, bone marrow; CI, confidence interval; CR1, first complete remission; CR2, second complete remission; GVHD, graft-versus-host disease; HSCT, hematopoietic stem cell transplantation; *n*, number; PB, peripheral blood; WBC, white blood cells. *GVHD was classified as the revised NIH consensus criteria (*Biol Blood Marrow Transplant* **2015**; *21*; 389–401).

Table S3. Multivariate analysis to reveal an impact of *KIT* or D816 *KIT* mutations on survival outcomes

**Model #1**	n	**Relapse**		**Non-Relapse Mortality**		**Disease-Free Survival**		**Overall Survival**	
		**HR (95% CI)**	***P* Value**	**HR (95% CI)**	***P* Value**	**HR (95% CI)**	***P* Value**	**HR (95% CI)**	***P* Value**
KIT mutations									
Unmutated	96	1		1		1		1	
Mutated	70	3.08 (1.18–7.0)	0.021	1.77 (0.76–4.09)	0.184	3.05 (1.54–6.04)	0.001	3.33 (1.58–7.02)	0.002
Age at HSCT, years	166			1.02 (0.99–1.06)	0.164	1.02 (0.99–1.04)	0.157	1.03 (1.00–1.05)	0.046
Disease state									

CR1	75	1				1		1	
CR2	79	7.28 (1.88–28.1)	0.004			5.33 (2.0–14.13)	0.001	4.64 (1.61–13.32)	0.004
Transplant type									
Allo-HSCT	112			1		1		1	
Auto-HSCT	54			0.12 (0.16–0.92)	0.041	1.08 (0.49–2.38)	0.849	0.84 (0.34–2.05)	0.699

**Model #2**	**n**	**Relapse**		**Non-Relapse Mortality**		**Disease-Free Survival**		**Overall Survival**	
		**HR (95% CI)**	***P* Value**	**HR (95% CI)**	***P* Value**	**HR (95% CI)**	***P* Value**	**HR (95% CI)**	***P* Value**
D816V *KIT* mutation									
Unmutated	145	1				1		1	
Mutated	21	5.60 (2.27–13.8)	<0.001			2.95 (1.49–5.80)	0.002	2.58 (1.25–5.31)	0.010
Age at HSCT, years	166			1.03 (0.99–1.06)	0.158	1.02 (0.99–1.04)	0.106	1.03 (1.00–1.05)	0.031
Disease state									
CR1	75	1				1		1	
CR2	79	6.41 (1.79–23.0)	0.004			3.85 (1.54–9.61)	0.004	3.12 (1.16–8.40)	0.024
Transplant type									
Allo-HSCT	112			1		1		1	
Auto-HSCT	54			0.10 (0.01–0.73)	0.023	0.90 (0.42–1.94)	0.788	0.65 (0.27–1.56)	0.336

Abbreviations: Allo-HSCT, allogeneic HSCT; Auto-HSCT, autologous HSCT; CI, confidence interval; CR1, first complete remission; CR2, second complete remission; HR, Hazard ratio; HSCT, hematopoietic stem cell transplantation; *n*, number.

Table S4. Impact of transplant type on kinetics of *RUNX1-RUNX1T1* transcript levels*.

**Variables**	***n***	**Time Range of the Determinations**	**Non-Evaluable Patients**	**Log_{10} Transformed Transcript Levels**			**Log Reduction**		
				**Allo-HSCT**	**Auto-HSCT**	***p***	**Allo-HSCT**	**Auto-HSCT**	***p***

Pre-HSCT	166	Between days 47 and 14 before HSCT	NA	−3.5 ± 0.14	−4.1 ± 0.19	0.023	−4.20 ± 0.14	−4.78 ± 0.18	0.015
1 month after HSCT	83	Between days 25 and 35 after HSCT	NRM < 1 month (*n* = 1)<br>Relapse <1 month (*n* = 0)<br>MRD not performed (*n* = 82)	−4.24 ± 0.17	−5.13 ± 0.39	0.046	−4.87 ± 0.17	−5.88 ± 0.37	0.024
3 months after HSCT	102	Between days 84 and 99 after HSCT	NRM < 3 months (*n* = 5)<br>Relapse <3 months (*n* = 0)<br>MRD not performed (*n* = 59)	−4.84 ± 0.15	−5.03 ± 0.37	0.636	−5.50 ± 0.15	−5.76 ± 0.37	0.504

Abbreviations: HSCT, hematopoietic stem cell transplantation; *n*, number; NA, non-applicable; NRM, non-relapse mortality; MRD, measurable residual disease. **RUNX1-RUNX1T1* transcript levels were normalized with respect to the number of *ABL1* transcripts and expressed as copy numbers per 10^5 copies of *ABL1*. Data were expressed as mean ± SEM.

Table S5. Sensitivity and specificity of *RUNX1-RUNX1T1* MRD positive patients defined by various cutoffs at each time points*.

Various Cutoffs at Each Time Point of MRD Assessment	MRD Positive Patents (%)	Relapsed Patients Among MRD Positive Patients	Sensitivity	MRD Negative Patents (%)	Non-Relapsed Patients Among MRD Negative Patients	Specificity
Pre-HSCT (*n* = 166)						
1000 copies	15 (9)	5	33%	151 (91)	135	89%
500 copies	21 (13)	7	33%	145 (87)	131	90%
250 copies	31 (19)	8	26%	135 (81)	122	90%
100 copies	53 (32)	11	21%	113 (68)	103	91%
50 copies	73 (44)	16	22%	93 (56)	88	95%
10 copies	111 (67)	18	16%	55 (33)	52	95%
0 copies	130 (78)	21	16%	36 (22)	36	100%
3 log reduction	21 (13)	8	38%	145 (87)	132	91%
4 log reduction	71 (43)	15	21%	95 (57%)	89	94%

1 month after HSCT (*n* = 83)						
1000 copies	3 (4)	2	67%	80 (96)	70	88%
500 copies	4 (5)	2	50%	79 (95)	69	87%
250 copies	7 (8)	3	43%	76 (92)	67	88%
100 copies	20 (24)	7	35%	63 (76)	58	92%
50 copies	28 (34)	8	29%	55 (66)	51	93%
10 copies	43 (52)	10	23%	40 (48)	38	95%
0 copies	50 (60)	11	22%	33 (40)	32	97%
3 log reduction	4 (5)	3	75%	79 (95)	70	89%
4 log reduction	27 (33)	8	30%	56 (68)	52	93%
3 months after HSCT (*n* = 102)						
1000 copies	4 (4)	3	75%	98 (96)	90	92%
500 copies	5 (5)	4	80%	97 (95)	90	93%
250 copies	8 (8)	6	75%	94 (92)	89	95%
100 copies	13 (13)	6	46%	89 (87)	84	94%
50 copies	17 (17)	7	41%	85 (83)	81	95%
10 copies	30 (29)	9	30%	72 (71)	70	97%
0 copies	45 (44)	10	22%	57 (56)	56	98%
3 log reduction	6 (6)	5	83%	96 (94)	90	94%
4 log reduction	14 (14)	7	50%	88 (86)	84	96%

Abbreviations: HSCT, hematopoietic stem cell transplantation; n, number: MRD, measurable residual disease. **RUNX1-RUNX1T1* transcript levels were normalized with respect to the number of *ABL1* transcripts and expressed as copy numbers per 10^5 copies of *ABL1*.

Table S6. Multivariate analysis to reveal an impact of *RUNX1-RUNX1T1* quantification at each time point on survival outcomes.

**Model #1 (*n* = 166)**	**n**	**Relapse**		**Disease-Free Survival**		**Overall Survival**	
		**HR (95% CI)**	***P* Value**	**HR (95% CI)**	***P* Value**	**HR (95% CI)**	***P* Value**
***RUNX1-RUNX1T1* Levels at Pre-HSCT**							
≥ 3 log reduction	145	1		1		1	

< 3 log reduction	21	6.62 (2.67–16.39)	<0.001	2.92 (1.48–5.75)	0.002	2.97 (1.45–6.08)	0.003
Age at HSCT, years	166			1.02 (0.99–1.04)	0.174	1.03 (1.00–1.05)	0.048
Disease state							
CR1	156	1		1		1	
CR2	10	5.76 (1.63–20.39)	0.007	3.12 (1.28–7.59)	0.012	2.60 (0.99–6.82)	0.052
Transplant type							
Allo-HSCT	112			1		1	
Auto-HSCT	54			0.79 (0.37–1.67)	0.535	0.60 (0.25–1.41)	0.241

Model #2 (*n* = 83)	*n*	Relapse		Disease-Free Survival		Overall Survival	
		**HR (95% CI)**	***P* Value**	**HR (95% CI)**	***P* Value**	**HR (95% CI)**	***P* Value**
RUNX1-RUNX1T1 levels at 1 month after HSCT							
≥ 3 log reduction	79	1		1		1	
< 3 log reduction	4	13.44 (3.39–53.38)	<0.001	3.33 (0.94–11.81)	0.062	4.33 (1.22–15.40)	0.023
Age at HSCT, years	83			1.00 (0.98–1.03)	0.764	1.02 (0.99–1.05)	0.249
Disease state							
CR1	75	1		1		1	
CR2	8	6.20 (1.59–24.2)	0.009	2.92 (1.08–7.91)	0.035	2.35 (0.79–7.04)	0.127
Transplant type							
Allo-HSCT	70			1		1	
Auto-HSCT	13			0.24 (0.03–1.80)	0.164	0 (0–3.165E+244)	0.965

Model #3 (*n* = 102)		Relapse		Disease-Free Survival		Overall Survival	
		**HR (95% CI)**	***P* Value**	**HR (95% CI)**	***P* Value**	**HR (95% CI)**	***P* Value**

RUNX1-RUNX1T1 levels at 3 months after HSCT							
≥ 3 log reduction	96	1		1		1	
< 3 log reduction	6	36.36 (9.91–133.37)	<0.001	6.49 (2.14–19.75)	0.001	4.69 (1.40–15.71)	0.012
Age at HSCT, years	102			1.03 (0.99–1.06)	0.152	1.03 (0.99–1.07)	0.074
Disease state							
CR1	94	1		1		1	
CR2	8	6.02 (1.15–31.45)	0.033	3.53 (1.17–10.65)	0.025	2.67 (0.77–9.29)	0.123
Transplant type							
Allo-HSCT	86			1		1	
Auto-HSCT	16			0.60 (0.14–2.63)	0.502	0.56 (0.13–2.46)	0.446

Abbreviations: Allo-HSCT, allogeneic HSCT; Auto-HSCT, autologous HSCT; CI, confidence interval; CR1, first complete remission; CR2, second complete remission; HSCT, hematopoietic stem cell transplantation; *n*, number.
